# Ephedrine Attenuates LPS‐Induced Acute Lung Injury in Mice by Inhibiting OTUB1 and Promoting K48 Ubiquitination of HIF1α

**DOI:** 10.1111/jcmm.70598

**Published:** 2025-05-19

**Authors:** Bo Zhou, Keke Zhao, Jiahui Xue, Fangling Zhou, Jin‐ao Duan, Yang Niu, Hanqing Wang

**Affiliations:** ^1^ College of Pharmacy, Key Laboratory of Ningxia Minority Medicine Modernization, Ministry of Education Ningxia Medical University Yinchuan Ningxia China; ^2^ National and Local Collaborative Engineering Center of Chinese Medicinal Resources Industrialization and Formulae Innovative Medicine Nanjing University of Chinese Medicine Nanjing China; ^3^ Key Laboratory of Protection, Development and Utilization of Medicinal Resources in Liupanshan Area, Ministry of Education Ningxia Medical University Yinchuan Ningxia China; ^4^ Ningxia Regional Characteristic Traditional Chinese Medicine Collaborative Innovation Center co‐Constructed by the Province and Ministry, Ningxia Engineering and Technology Research Center for Modernization of Regional Characteristic Traditional Chinese Medicine Ningxia Medical University Yinchuan China

**Keywords:** acute lung injury, ephedrine, HIF1α, OTUB1

## Abstract

Acute lung injury (ALI) is a severe inflammatory lung disorder that requires effective therapeutic strategies. Ephedrine (EPH) is the main active component found in medicinal plants of the Ephedra genus and is commonly used to modulate inflammatory responses in various diseases. Hypoxia‐inducible factor 1‐alpha (HIF1α) is a subunit of hypoxia‐inducible factor 1 (HIF1), which plays a critical regulatory role in cellular responses under hypoxic conditions. Moreover, the degradation pathway of HIF1α is regulated by the deubiquitinase Ovarian Tumour Domain‐containing Ubiquitin Aldehyde Binding Protein 1 (OTUB1). The aim of this study is to investigate the therapeutic effects of EPH on ALI and its potential therapeutic mechanism. We utilised a lipopolysaccharide (LPS)‐induced ALI mouse model and employed various methods for evaluation. Ultimately, our research findings demonstrate that EPH exhibits anti‐ALI effects, with the involvement of HIF1α and OTUB1 in the pharmacological actions of EPH. In conclusion, our study results demonstrate that EPH exhibits anti‐ALI effects and exerts its protective effects through modulation of the OTUB1 and HIF1α pathways. Our research findings not only lay the foundation for expanding the medicinal applications of EPH but also provide direction for seeking improved treatment strategies for ALI.

**Abbreviations**
ALI: acute lung injury
bHLH‐PAS: basic helix–loop–helix/Per‐Arnt‐Sim
CAT: catalase
DAMPs: damage‐associated molecular patterns
DUBs: deubiquitinating enzymes
EPH: ephedrine
HIF1: hypoxia‐inducible factor 1
HIF1α: hypoxia‐inducible factor 1‐alpha
IL‐10: interleukin‐10
IL‐1R: interleukin‐1 receptors
LPS: lipopolysaccharide
MDA: malondialdehyde
MJD: Machado‐Joseph disease proteases
ODDD: oxygen‐dependent degradation domain
OTU: ovarian tumour proteases
OTUB1: ovarian tumour domain‐containing ubiquitin aldehyde binding protein 1
PTEN: phosphatase and tensin homologue
SOD: superoxide dismutase
TLRs: toll‐like receptors
TNF‐α: tumour necrosis factor α
UBP: ubiquitin‐specific peptidases
UPS: ubiquitin‐proteasome system
USP: ubiquitin‐specific proteases

## 1 Introduction

ALI is a pulmonary inflammatory disease characterised by a clinical syndrome caused by various extrapulmonary or intrapulmonary factors [1]. ALI is characterised by injury to alveoli and pulmonary capillaries, resulting in impaired lung function [2]. Clinically, ALI presents with diffuse pulmonary infiltrates, refractory hypoxemia, and respiratory distress. Pathologically, it involves injury to pulmonary capillary endothelial cells and alveolar epithelial cells, diffuse alveolar and interstitial oedema, severe pulmonary inflammation and disruption of the alveolar‐capillary barrier [3, 4]. LPS, an outer membrane component of Gram‐negative bacteria, can induce severe inflammatory reactions [5]. LPS‐induced severe pulmonary inflammation can cause lung hypoxia, leading to the development of ALI [6]. The key pathophysiological processes involved in ALI are inflammation, increased vascular permeability, oxidative stress and cellular apoptosis [7]. Due to the incomplete understanding of the mechanisms underlying ALI and the need for further development and improvement of therapeutic drugs, exploring novel compounds is necessary to fill this gap [8]. Compounds extracted from herbal medicines have shown potential in alleviating abnormal inflammatory responses and mitigating lung diseases [9, 10, 11, 12]. Previous studies have indicated that EPH can alleviate lung injury induced by certain external factors, but the precise mechanism of its therapeutic effects remains unknown [13].

The pharmacologically active component found in medicinal plants of the Ephedra genus is EPH [14]. EPH is a commonly used adrenergic agonist and has been shown to regulate inflammatory responses in various diseases such as acute liver failure, allergic asthma and ischemic stroke [15, 16, 17, 18]. For instance, EPH hydrochloride has been demonstrated to exert protective effects in mice stimulated with LPS by promoting the secretion of anti‐inflammatory cytokines such as interleukin (IL)‐10 and reducing the release of pro‐inflammatory factors like tumour necrosis factor (TNF)‐α [19]. Furthermore, EPH has been shown to mitigate nephrotoxicity and hepatotoxicity by attenuating oxidative damage and genetic toxicity in mice treated with cisplatin [20]. Additionally, EPH exhibits significant reduction of histopathological damage and lung indices in mice with chronic obstructive pulmonary disease by inhibiting oxidative damage, inflammation and cell apoptosis through blocking endoplasmic reticulum stress [21].

HIF1 mediates the adaptive response of tissues to changes in oxygen levels within the body [22]. Under normal oxygen levels, HIF1α undergoes hydroxylation, followed by ubiquitination by the Von Hippel–Lindau ubiquitin ligase, targeting HIF1/2α for ubiquitin‐proteasome degradation [23]. Under low oxygen conditions, the activation or loss of certain proteins in the cell can lead to an increase in HIF1α levels. For example, loss of phosphatase and tensin homologue (PTEN) or p53 function can increase HIF1α synthesis and stability [24, 25, 26]. Activated tyrosine kinases can stimulate HIF1α synthesis, and mTOR can also increase HIF1α stability [25, 27, 28]. Synthesised or stabilised HIF1α binds to HIF1β to form the active HIF1 complex, which subsequently influences various physiological functions in cells [29]. Low oxygen not only increases HIF1α levels in all cells, but it is also associated with reduced degradation of HIF1α [30]. The degradation of HIF1α is primarily mediated by the ubiquitin‐proteasome system (UPS) in most cases [31].

The UPS is an intracellular protein degradation system [32]. Its main function is to identify, tag and degrade unnecessary, abnormal, or excessively modified proteins. The core of UPS is the ubiquitin protein, and the central process is ubiquitination [33]. Interestingly, the process of ubiquitination is reversible. Deubiquitinating enzymes (DUBs) can remove ubiquitin molecules from proteins that have already been ubiquitinated [34]. DUBs can be classified into several families based on their structure and mechanism, including ubiquitin‐specific peptidases (UBP) (), ubiquitin‐specific proteases (USP) (), ovarian tumour proteases (OTU) (), Machado‐Joseph disease proteases (MJD) (), and others [35]. Each family of DUBs possesses specific structures and catalytic mechanisms [36]. OTUB1 belongs to the OTU family and possesses a conserved OTU domain, which exhibits deubiquitinating activity [37]. OTUB1 primarily exerts its deubiquitinating function by binding to ubiquitin and cleaving ubiquitin chains [38]. It exhibits specificity towards various types of ubiquitin chains, including K48 and K63 chains [39]. Previous studies have shown that OTUB1 is involved in the K48‐linked polyubiquitination of HIF1α [40]. This observation prompted us to investigate whether OTUB1 and HIF1α mediate the potential mechanism of EPH's therapeutic efficacy in the treatment of ALI.

ALI can be triggered through multiple pathways. In some of these pathways, damage‐associated molecular patterns (DAMPs) released from dead cells serve as danger signals that activate and recruit immune cells by binding to various receptors, such as Toll‐like receptors (TLRs) and interleukin‐1 receptors (IL‐1R), thereby initiating pro‐inflammatory pathways [39]. Other studies suggest that chemical injury may induce oxidative damage to lung cells, leading to the activation of intracellular kinases [41]. Regardless of the aetiology of ALI, its pathogenesis is fundamentally related to uncontrolled inflammatory responses. In this study, we found that OTUB1 plays a crucial role in an LPS‐induced ALI mouse model. Furthermore, we discovered that EPH can alleviate LPS‐induced ALI by inhibiting OTUB1 expression, thereby promoting K48 ubiquitination and degradation of HIF1α.

## 2 Materials and Methods

### 2.1 Animals

75 male C57BL/6 mice (6–8 weeks old) were purchased from Sibeifu (Suzhou) Biotechnology Co. Ltd. (Animal Qualification Certificate: SCXK (Su) 2022‐0006). The mice were housed in a controlled indoor environment with a temperature of 23 ± °C and humidity of 40%–60% with a 12‐h light–dark cycle.

### 2.2 AAV‐OTUB1 Adenovirus Transfection in Mice

The dose of AAV‐OTUB1 adenovirus transfection in mice was 1.5 × 10^12^ viral genome copies/mouse, administered via tail vein injection. The mice were subjected to modelling and drug treatment 2 weeks after transfection.

### 2.3 ALI Model

After 1 week of adaptive feeding, the mice were randomly divided into the sham group (Sham) (*n* = 15), model group (LPS) (*n* = 15), dexamethasone group (Dex, 5 mg/kg) (*n* = 15), Eph low dose group (Eph‐L, 5 mg/kg) (*n* = 15) and Eph high dose group (Eph‐H, 10 mg/kg) (*n* = 15). Mice were given LPS 10 mg/kg by nasal infusion. The EPH group was administered once at 0 h, 2 h and 12 h after nasal instillation of LPS. Mice were sacrificed 24 h or 7 days post‐exposure for experimental studies and inflammation analysis. For survival analysis, mice were given a lethal dose (25 mg/kg) of LPS challenge to induce severe ALI. The mouse survival in each group was recorded every day for 7 days.

### 2.4 Lung Histopathological Testing

The excised lung tissue was fixed in 4% paraformaldehyde at room temperature for 24 h, followed by dehydration using an automatic dehydrator and embedding in paraffin. Subsequently, the tissue was sectioned into 4 μm thick slices using a rotary microtome and placed on glass slides. The slides were baked in an oven at 60°C for 1 h to ensure proper adhesion of the sections. The paraffin was removed by two washes with xylene, each for 10 min. The sections were then rehydrated through a graded series of ethanol (100%, 95%, 80% and 70%), with each step lasting 5 min. The slides were washed three times with phosphate‐buffered saline (PBS), each for 5 min. Staining commenced with haematoxylin solution for 3–5 min, followed by rinsing with tap water for 1–2 min. Differentiation was achieved using 70% ethanol containing 0.25% HCl for 1 min, and then treated with 2% sodium bicarbonate for 5–10 min, followed by a 2‐min rinse under running tap water. Next, the slides were stained with eosin for 1–3 min, followed by a wash with tap water for 30–60 s. The sections were dehydrated through a series of ethanol concentrations and then cleared in xylene for 4 min before being mounted with neutral resin. Finally, the HE‐stained sections were observed using an optical microscope.

### 2.5 Immunofluorescent Staining of OTUB1 and HIF1α in Lung Tissue

Lung tissue fixed in 4% paraformaldehyde was subjected to dehydration and paraffin embedding. The tissue was sectioned into 4 μm thick slices using a rotary microtome. The slices were deparaffinised with xylene and rehydrated through a gradient of ethanol. Antigen retrieval was performed using sodium citrate buffer for 10 min, followed by incubation with 3% hydrogen peroxide at room temperature for 10 min. The sections were then blocked with goat serum for 30 min and incubated overnight at 4°C with primary antibodies against OTUB1 (Abcam, ab270959, diluted 1:200) and HIF1α (Thermo Fisher, MA1‐516, diluted 1:200). The next day, the primary antibodies were discarded, and the sections were incubated at room temperature for 1 h with fluorescent secondary antibodies (Abcam, ab150077, diluted 1:1000; ab150116, diluted 1:1000), followed by mounting with neutral gum. Microscopic examination and image acquisition were performed for analysis.

### 2.6 Bronchoalveolar Lavage Fluid Collection

After euthanasia, the thorax and trachea of mice were exposed, the main trachea and the right pulmonary bronchus were ligated, and the left lung of the mice was irrigated with 0.4 mL pre‐cooled PBS for three times. BALF was centrifuged at 350 g for 5 min at 4°C. The supernatant was collected and stored in 80°C freezer until use.

### 2.7 Enzyme Linked Immunosorbent Assay

The expression levels of pro‐inflammatory cytokines IL‐6, IL‐1β and TNF‐α in BALF were measured by the enzyme linked immunosorbent assay (ELISA) assay. Thaw the BALF samples and prepare the wash solution, dilution solution, and standard solutions according to the instructions provided by the kit. Add 100 μL of different concentrations of standard solutions and experimental samples into the corresponding wells. Seal the reaction wells with adhesive film and incubate at room temperature for 2 h. Remove the liquid from the wells, wash the plate using a wash bottle, and then add 100 μL of enzyme‐labelled detection antibody to each well. Incubate at room temperature for 2 h, followed by another washing step. Then, add 200 μL of substrate solution to each well and incubate in the dark at room temperature for 30 min. Finally, add 50 μL of stop solution to each well, and measure the absorbance at 450 nm using a microplate reader.

### 2.8 Western Blot

The fresh lung tissues were washed twice with PBS solution, followed by homogenisation using a high‐throughput tissue homogeniser in RIPA lysis buffer containing 1% PMSF and 2% phosphatase inhibitors. The homogenate was centrifuged at 4°C and 12400 g for 20 min, and the supernatant was collected. The protein concentration in the supernatant was determined using a BCA protein assay kit according to the manufacturer's instructions. Equal amounts of protein from each sample were separated by 12% sodium dodecyl sulphate‐polyacrylamide gel electrophoresis (SDS‐PAGE) and transferred onto a polyvinylidene difluoride (PVDF) membrane. After incubation with primary and secondary antibodies followed by washing, protein bands were detected using the Tanon gel imaging system, and the grayscale values of the protein bands were analysed using Image J software.

### 2.9 Co‐Immunoprecipitation

The fresh lung tissues were washed twice with PBS solution and lysed with Co‐IP cell lysis buffer. The lysate was centrifuged at 4°C and 12,000 rpm for 10 min, and the supernatant was collected for protein quantification. The lysate was then incubated overnight at 4°C with anti‐HIF1α antibody (Abcam, ab308433, diluted 1:30) or anti‐IgG antibody (CST, 3420S, diluted 1:20). The mixture was precipitated using 50% protein A/G agarose beads solution and gently rocked overnight at 4°C, followed by centrifugation to collect the precipitated bound complex. The pellet was denatured at 100°C for 10 min with 1× Loading buffer. Subsequent Western blot experiments were performed.

### 2.10 Quantitative Real‐Time PCR (QRT‐PCR)

Using Trizol reagent (Sigma, Cat. No. T9424), total RNA was extracted from mouse lung tissue samples following the manufacturer's instructions. The integrity and purity of RNA were evaluated using a spectrophotometer. The UEIris II RT‐PCR System for First‐Strand cDNA Synthesis kit (UE, Cat. No. R2028) was employed for one‐strand cDNA synthesis, wherein the RNA samples were treated with dsDNase to remove genomic DNA contamination. PCR reactions were performed using the qPCR SYBR Green Master Mix (High Rox Plus) kit (Yeasen, Cat. No. 11203ES03). Ct values were obtained for each target gene and reference gene. The relative gene expression levels were calculated using the 2^−ΔΔCt^ method. The sequences of primers for qRT‐PCR are as Table 1.

**TABLE 1:** Primer sequences for qRT‐PCR.

Primer name	5′‐3′
Gapdh‐F	GCCTCCTCCAATTCAACCCT
Gapdh‐R	CTCGTGGTTCACACCCATCA
OTUB1‐F	GCTGTGCAGAATCCTCTGGT
OTUB1‐R	AAGCCAAACGCTCGGTAGAA

### 2.11 Detection of Myeloperoxidase Enzyme Activity in Lung Tissue

Myeloperoxidase (MPO) enzyme activity was detected by the MPO kit (Nanjing Jiancheng Bioengineering Institute, A044‐1‐1).

### 2.12 Statistical Analysis

The experimental data were presented as Mean ± SD. Statistical analysis was performed using GraphPad Prism 8 software, employing one‐way analysis of variance (ANOVA) and Dunnett's multiple comparisons test. *p < 0.05 indicates a statistically significant difference between the data, while **p < 0.01 indicates a highly significant difference.

## 3 Results

### 3.1 EPH Exhibits Anti‐ALI Effects

**FIGURE 1:**
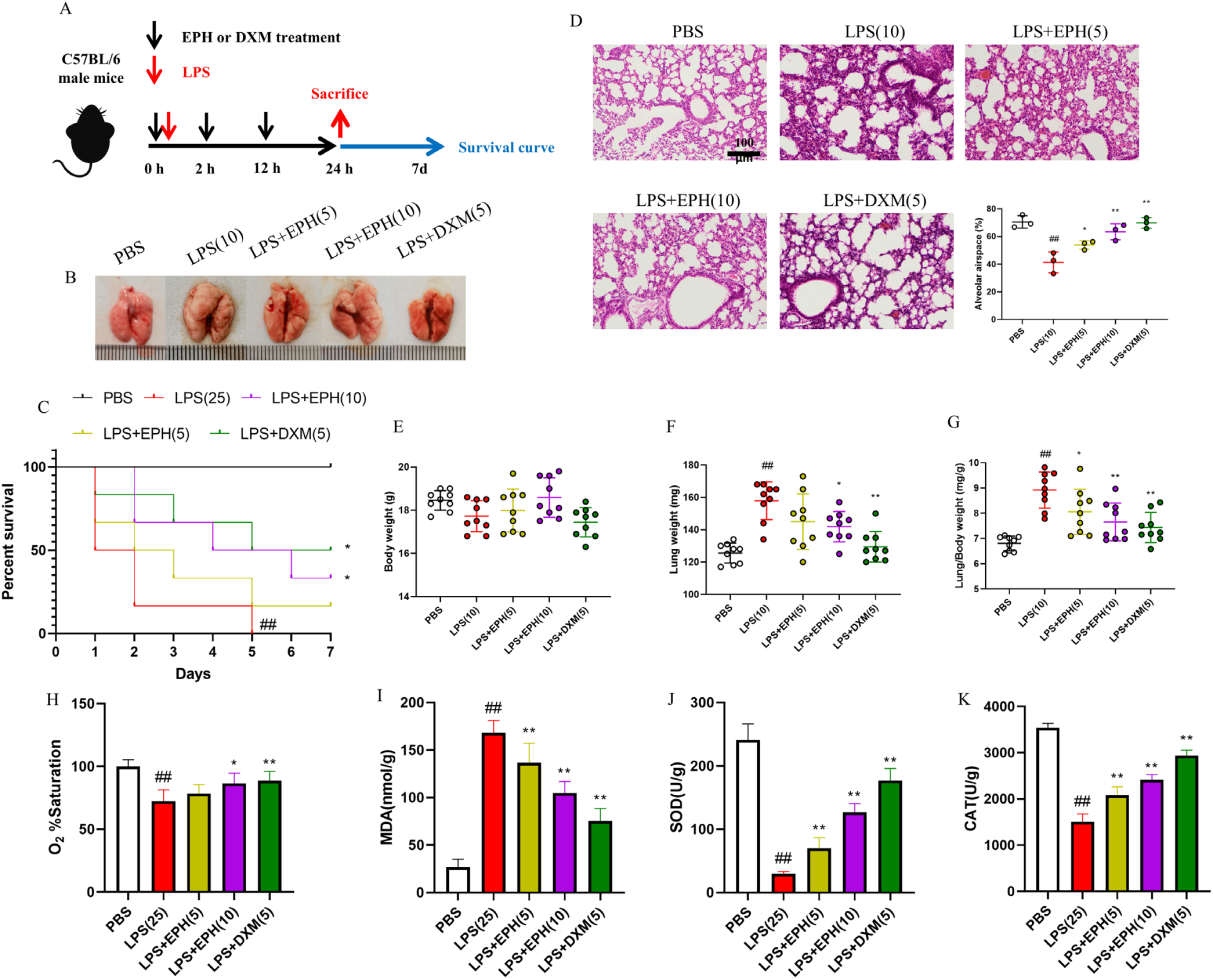
EPH exhibits anti‐ALI effects. (A) Animal experimental procedure. (B) Comparison of lung tissues before and after drug administration. (C) Animal survival rate. (D) Pathological examination of lung tissues and statistical analysis of pulmonary alveolar interstitial spaces, H&E staining. (E) Mouse body weight. (F) Mouse lung weight. (G) Lung tissue organ index. (H–K) Determine the levels of O_2_% saturation, MDA, SOD and CAT as oxidative indicators in lung tissue. ## indicates *p* < 0.01, significantly different from the blank group (PBS); * indicates *p* < 0.05, significantly different from the model group (LPS); ** indicates *p* < 0.01, significantly different from the model group (LPS).

To investigate the role of EPH in treating ALI, we utilised a mouse model induced by LPS administration via the nasal route at a dosage of 10 mg/kg. Subsequently, we examined the survival rate of mice administered with LPS at a dosage of 25 mg/kg. Following LPS or PBS treatment, mice were orally administered EPH at 0, 2 and 12 h, with sacrifice at 24 h for survival rate analysis and subsequent experiments (Figure 1A). After sacrificing the mice and obtaining their lung tissues, we observed the overall morphological changes induced by EPH in LPS‐induced lung tissues. Following LPS stimulation, significant lung tissue swelling was observed compared to the PBS group. Encouragingly, EPH treatment alleviated lung tissue swelling (Figure 1B). In the survival rate test, as time progressed, the survival rate of mice in the model group exhibited a significant downward trend compared to the control group. However, this trend was reversed following EPH administration, with mice treated with 10 mg/kg EPH showing a higher reversal trend in survival rate compared to those treated with 5 mg/kg EPH (Figure 1C). Furthermore, to assess the impact of EPH on changes in pulmonary alveolar interstitial spaces, we conducted H&E staining to observe this pathological change in lung tissues. Data analysis revealed a reversal of the decrease in pulmonary alveolar interstitial spaces induced by LPS stimulation after EPH administration (Figure 1D). Additionally, to comprehensively evaluate the effects of EPH on lung tissues in ALI, we measured the lung tissue organ index of mice in each group. The results showed that compared to the control group, the lung tissue organ index of mice in the model group significantly increased. However, following oral administration of EPH, this index significantly decreased in a dose‐dependent manner (Figure 1E–G). Additionally, we assessed the hypoxia‐related parameters in each group of mice. The results indicated a significant decline in O2 saturation levels in the model group of mice subjected to LPS treatment, accompanied by an increase in malondialdehyde (MDA) levels, which suggests heightened oxidative stress and lipid peroxidation. Additionally, we observed a marked decrease in the activities of superoxide dismutase (SOD) and catalase (CAT) in the lungs of the model group, indicating impaired antioxidant defence mechanisms.

Conversely, upon administration of EPH, we noted a reversal of these indicators. Specifically, O2 saturation levels in the EPH‐treated mice significantly improved compared to the model group, suggesting enhanced oxygenation and respiratory function. Furthermore, MDA levels decreased substantially, indicating a reduction in oxidative stress and lipid damage following EPH treatment. Importantly, the activities of both SOD and CAT were elevated in the EPH‐treated group, reflecting a restoration of the antioxidant capacity in the lung tissue (Figure 1H–K). These findings collectively demonstrate that EPH exerts a protective effect against LPS‐induced ALI in mice by improving oxygen saturation and modulating oxidative stress markers, thereby prolonging the survival of mice with lung injury.

### 3.2 EPH Reduced the Release of Inflammatory Mediators in ALI Mouse Lung Tissues

**FIGURE 2:**
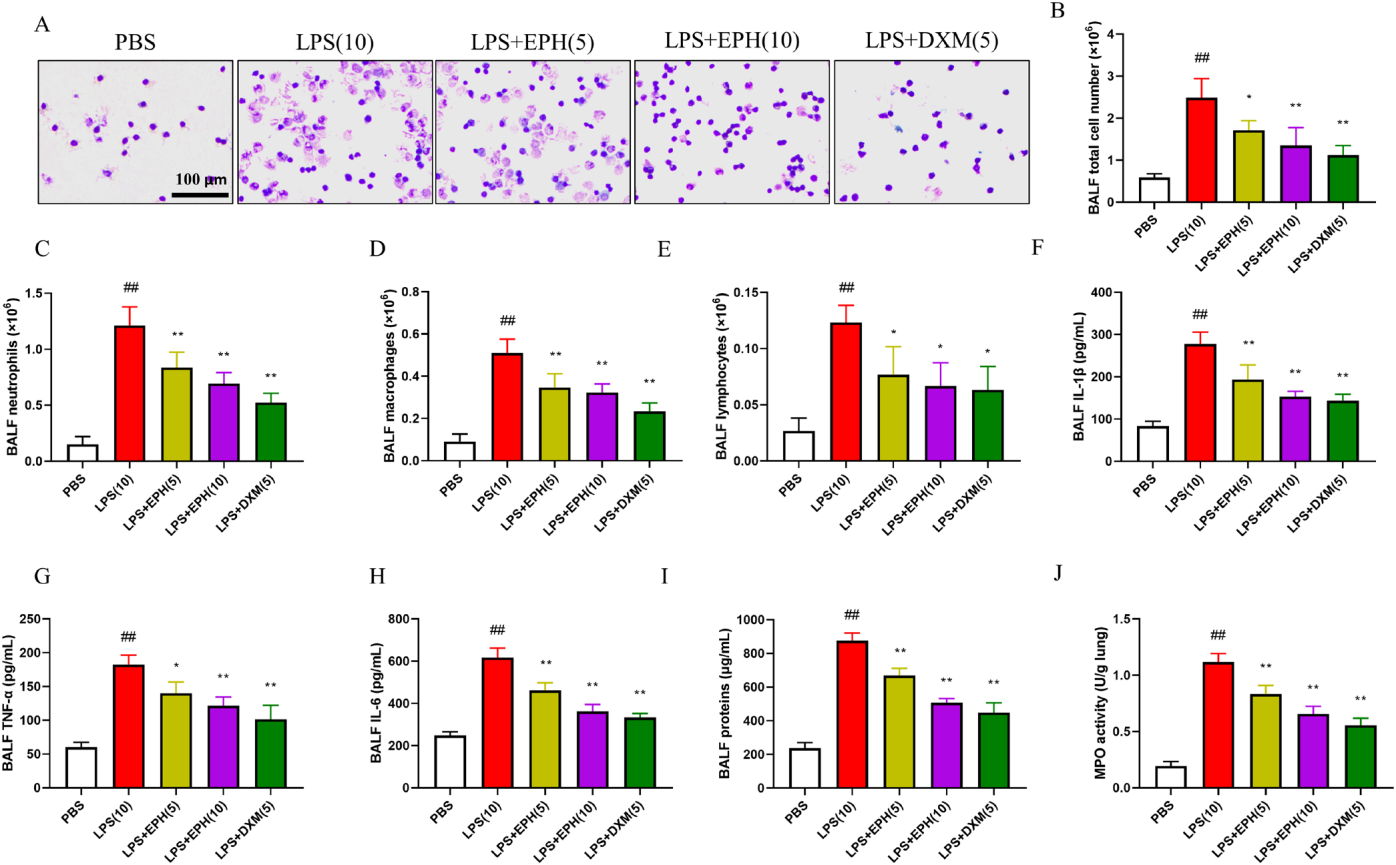
EPH reduced the release of inflammatory mediators in ALI mouse lung tissues. (A) Jimsa staining kit was used for BALF staining. (B) Total cell count in BALF. (C) Neutrophils in BALF. (D) Macrophage count in BALF. (E) Lymphocyte count in BALF. (F–H) ELISA was employed to detect the levels of pro‐inflammatory factors IL‐6, IL‐1β, TNF‐α in BALF. (I) Total protein content in BALF. (J) Measurement of MPO enzyme activity in lung tissue. ## indicates *p* < 0.01, indicating extremely significant difference compared to the blank group (PBS); * indicates *p* < 0.05, indicating significant difference compared to the model group (LPS); ** indicates *p* < 0.01, indicating extremely significant difference compared to the model group (LPS).

To investigate whether EPH is involved in suppressing inflammation, thereby protecting mice from ALI, we measured various cytokines in bronchoalveolar lavage fluid (BALF) from each group of mice (Figure 2A). Following LPS treatment, the number of infiltrating inflammatory cells in BALF significantly increased (Figure 2B). Additionally, the numbers of neutrophils (Figure 2C), macrophages (Figure 2D) and lymphocytes (Figure 2E) in BALF also significantly increased. However, after EPH treatment, the counts of these immune cells significantly decreased, indicating that EPH could alleviate the dramatic increase in pulmonary immune cells during ALI. Furthermore, in another experiment, we examined the expression of inflammatory cytokines in BALF, and data analysis similarly showed that EPH could downregulate the expression levels of IL‐1β (Figure 2F), TNF‐α (Figure 2G) and IL‐6 (Figure 2H), three inflammatory mediators. From a broader perspective, we observed the total protein content in BALF to understand the effect of EPH on vascular permeability. The results showed that EPH reduced vascular permeability compared to the model group (Figure 2I). Since MPO is mainly released by neutrophils and is an important indicator to evaluate the degree of neutrophil infiltration in lung tissue, indirectly reflecting the severity and extent of lung tissue damage, we found that LPS indeed increased the MPO content in lung tissue compared to the control group. Similarly, this situation was reversed after EPH administration, with a subsequent decrease in MPO levels (Figure 2J). Overall, these data indicate that EPH can alleviate the inflammatory response induced by LPS‐induced ALI, and higher doses of EPH have a better inhibitory effect on inflammatory cytokine secretion.

### 3.3 EPH Reduced the Expression of OTUB1 and HIF1α in Lung Tissue of Mice With ALI

**FIGURE 3:**
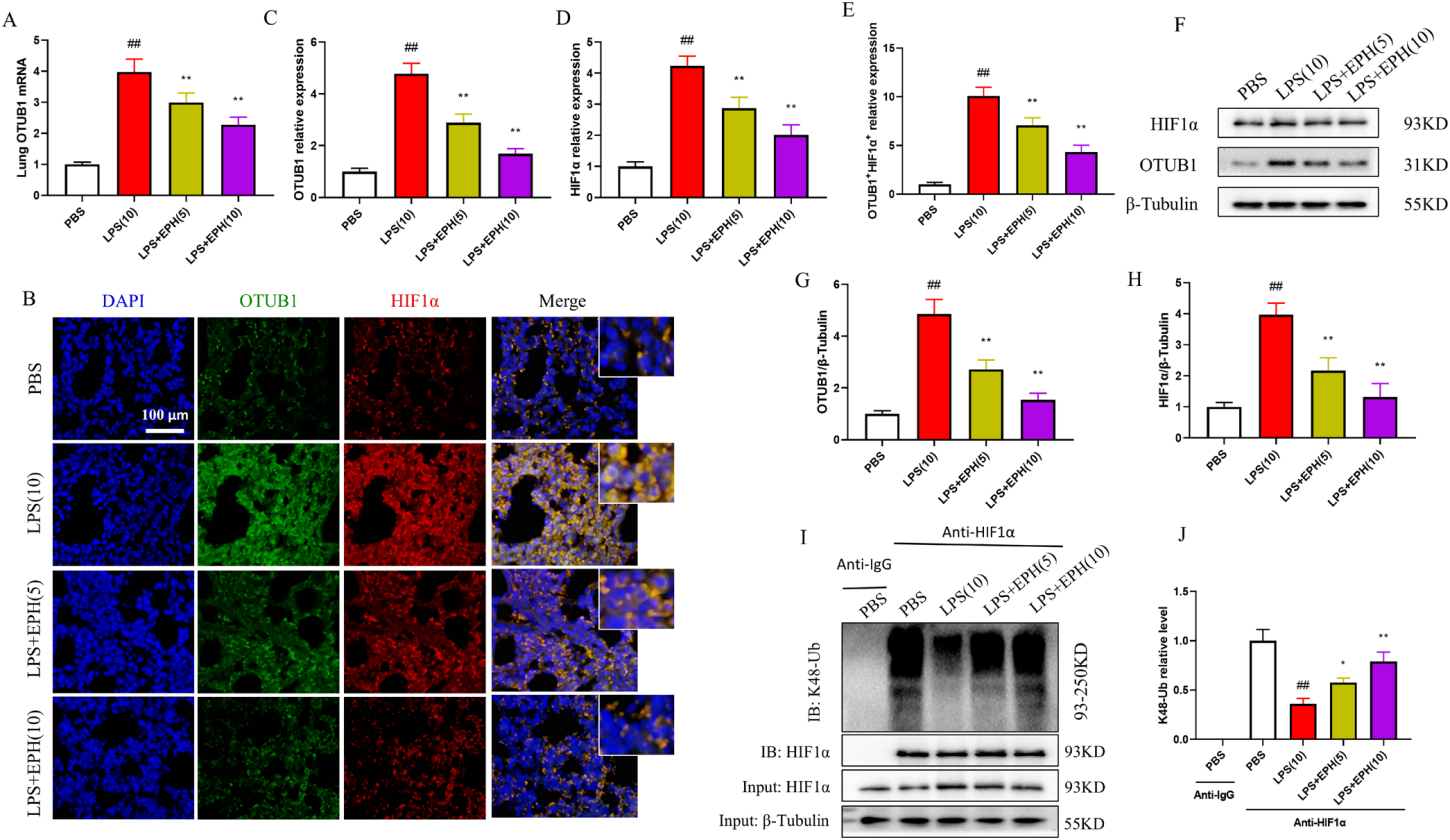
EPH reduced the expression of OTUB1 and HIF1α in lung tissue of mice with ALI. (A) Lung OTUB1 mRNA expression level. (B) Double immunofluorescence staining of OTUB1 and HIF1α. (C) Expression level of OTUB1 in lung tissue. (D) Expression level of HIF1α in lung tissue. (E) Data analysis of double immunofluorescence staining of OTUB1 and HIF1α. (F) Immunoblotting detection of OTUB1 and HIF1α expression in lung tissue. (G) Expression status of OTUB1 in lung tissue. (H) Expression status of HIF1α in lung tissue. (I) Immunoprecipitation to detect the ubiquitination level of HIF1α. (J) Quantitative analysis of HIF1α ubiquitination level. ## indicates *p* < 0.01, significantly different from the control group (PBS); * indicates *p* < 0.05, significantly different from the model group (LPS); ** indicates *p* < 0.01, significantly different from the model group (LPS).

Based on previous literature, we understand that OTUB1 can modulate HIF1α expression levels through non‐canonical ubiquitination inhibition, potentially stabilising HIF1α protein under hypoxic conditions [40]. Therefore, we investigated the effect of EPH on the protein expression levels of OTUB1 and HIF1α in lung tissue of mice with ALI. Data analysis revealed that compared to the control group, mice with LPS‐induced ALI exhibited a significant increase in OTUB1 mRNA (Figure 3A), as well as a significant elevation in the protein expression levels of OTUB1 and HIF1α in lung tissue (Figure 3C,D). However, intriguingly, the presence of EPH led to a contrasting expression trend in OTUB1 mRNA, OTUB1 protein and HIF1α protein. This result prompted us to speculate that EPH may regulate OTUB1 and HIF1α protein expression, potentially mediating its effects in ALI in mice. This speculation was validated in double immunofluorescence staining of OTUB1 and HIF1α (Figure 3B), where visualisation of the data showed an increase in co‐expression of OTUB1 and HIF1α in lung tissue of mice with LPS‐induced ALI, which was reversed by EPH (Figure 3E). Furthermore, considering the phenomenon of post‐translational protein modification of HIF1α and the fact that OTUB1 is a deubiquitinase known to inhibit K48 ubiquitination of HIF1α [40], we examined the expression of OTUB1 protein and HIF1α protein with EPH treatment using protein immunoblotting (Figure 3F). The data revealed that compared to the model group where both OTUB1 and HIF1α protein expressions increased synchronously, EPH treatment significantly reduced the levels of these two proteins (Figure 3G,H). This result validated our previous speculation. Subsequently, to further ascertain whether EPH could directly affect OTUB1‐mediated degradation of HIF1α protein through K48 ubiquitination, we investigated this hypothesis through immunoprecipitation (Figure 3I). Analysis showed that compared to the control group, K48 ubiquitination of HIF1α was significantly decreased in the model group, but EPH promoted the K48 ubiquitination degradation of HIF1α protein (Figure 3J). These findings suggest that in ALI, EPH may play a critical role in the OTUB1‐mediated K48 ubiquitination of HIF1α.

### 3.4 Overexpression of OTUB1 Blocks the Protective Effect of EPH Against ALI

**FIGURE 4:**
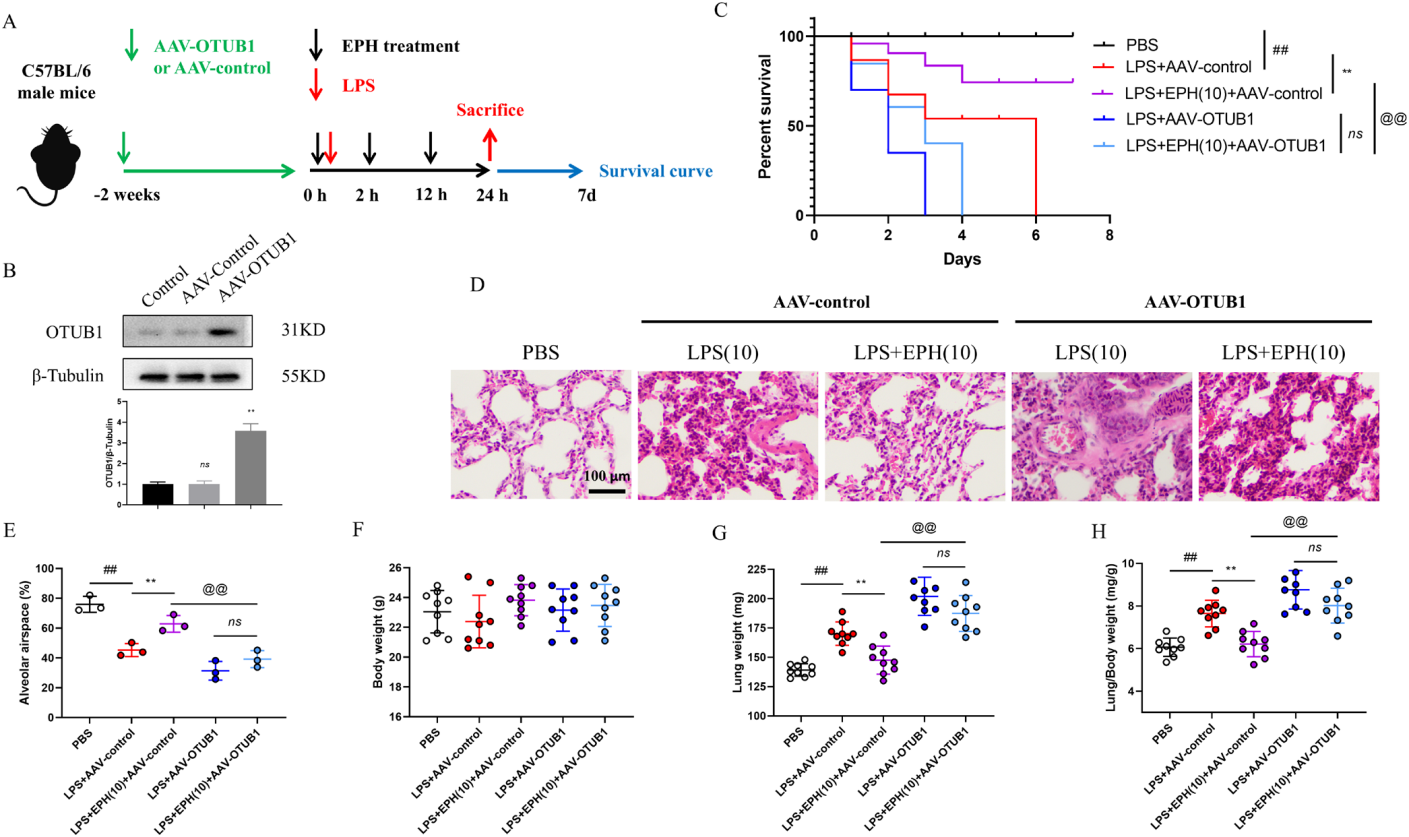
Overexpression of OTUB1 blocks the protective effect of EPH against ALI. (A) Animal experimental procedures. (B) Detection of OTUB1 protein overexpression by AAV‐OTUB1 virus. (C) Analysis of survival rate test data for animals. (D) Histopathological examination of lung tissue, HE staining. (E) Alveolar interstitial space. (F) Mouse body weight. (G) Mouse lung weight. (H) Lung tissue organ index. ## indicates *p* < 0.01, significant difference compared to the blank group (PBS); ** indicates *p* < 0.01, significant difference compared to the LPS‐AAV‐control group; @@ indicates *p* < 0.01, significant difference compared to the LPS‐AAV‐control treatment group. ‘ns’ indicates no significant difference.

To further investigate the role of EPH in OTUB1‐mediated HIF1αk48 ubiquitination, we conducted subsequent experiments using AAV‐OTUB1 mice overexpressing OTUB1 in the lungs, along with a control group of AAV‐control mice (Figure 4A). We confirmed a significant increase in OTUB1 protein levels in lung tissue of AAV‐OTUB1 mice (Figure 4B). Similarly, we initially performed survival rate tests on each group of mice as described previously. The final data showed that compared to other groups, mice with high OTUB1 expression exhibited the lowest survival rate after LPS stimulation. Additionally, EPH administration did not significantly increase the survival rate of mice with high OTUB1 expression. However, under the same conditions, the survival rate of mice in the AAV‐control group significantly increased after LPS stimulation and EPH administration compared to AAV‐OTUB1 mice (Figure 4C). Furthermore, to comprehensively assess the impact of EPH and OTUB1 on ALI, we examined lung tissue histopathological changes (Figure 4D) and measured lung interstitial space (Figure 4E) and lung tissue organ indices (Figure 4F–H). The results showed that compared to the control group, AAV‐OTUB1 mice exhibited thicker alveolar walls, reduced interstitial space and increased lung tissue organ indices. Interestingly, this trend was not significantly reversed after EPH administration. However, in AAV‐control mice, EPH effectively reversed the aforementioned trends. In essence, these experimental results suggest that OTUB1 plays a crucial role in EPH‐mediated protection against ALI.

### 3.5 Overexpression of OTUB1 Hinders EPH‐Induced Regulation of HIF1α and Inflammation in ALI

**FIGURE 5:**
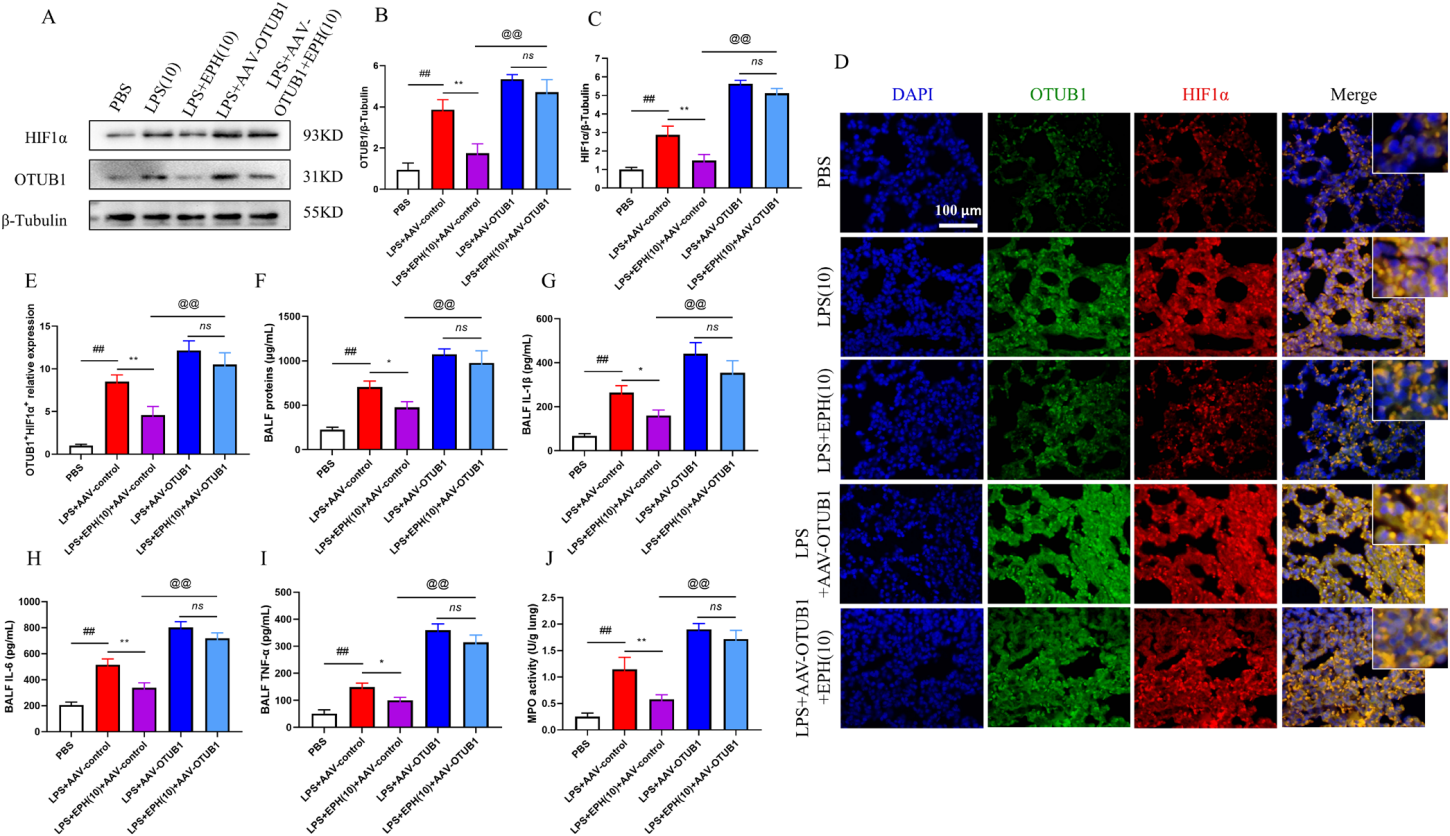
Overexpression of OTUB1 hinders EPH‐induced regulation of HIF1α and inflammation in ALI. (A) Immunoblotting analysis of the effects of OTUB1 overexpression on HIF1α and OTUB1 proteins. (B, C) Quantitative analysis of immunoblotting results. (D) Double immunofluorescence staining for HIF1α and OTUB1 proteins. (E) Analysis of OTUB1 and HIF1α expression levels. (F) Total protein content in BALF. (G) Expression level of IL‐1β. (H) Expression level of IL‐6. (I) Expression level of TNF‐α. (J) Measurement of MPO enzyme activity in lung tissue. ## indicates *p* < 0.01, significant difference compared to the blank group (PBS); ** indicates *p* < 0.01, significant difference compared to the LPS‐AAV‐control group; @@ indicates *p* < 0.01, significant difference compared to the LPS‐AAV‐control treatment group. ‘ns’ indicates no significant difference.

To further explore the potential mechanisms underlying the treatment of ALI with EPH, we examined the effects of OTUB1 overexpression on HIF1α and OTUB1 protein (Figure 5A). The results showed that compared to the control group, overexpression of OTUB1 enhanced the lung injury induced by LPS in mice. Additionally, the presence of OTUB1 led to a simultaneous increase in HIF1α expression and rendered the therapeutic efficacy of EPH ineffective in treating ALI (Figure 5B,C). Furthermore, we observed the co‐localization of OTUB1 and HIF1α using double immunofluorescence staining (Figure 5D). Co‐staining images of OTUB1 and HIF1α indicated that OTUB1 overexpression enhanced the immunofluorescent signal of HIF1α in the nucleus. Similarly, this trend remained unchanged after EPH administration (Figure 5E). Subsequently, we measured the total protein content in BALF, the expression levels of inflammatory cytokines IL‐1β, IL‐6, TNF‐α, and the activity of MPO enzyme in lung tissue for each group of mice. The results showed that compared to the control group, mice with ALI accompanied by OTUB1 overexpression exhibited significantly increased levels of total lung protein content, inflammatory cytokines and MPO. Furthermore, in mice with normal OTUB1 expression, EPH administration led to a significant decrease in inflammatory cytokines and MPO. However, this trend was not reversed after EPH administration in mice with OTUB1 overexpression (Figure 5F–J). These findings once again demonstrate the important role of OTUB1 and HIF1α in the process of EPH treatment for ALI.

## 4 Discussion

Our study identified the significant role of OTUB1 in mediating the protective effect of EPH on lung tissue against ALI. In our previous experiments, we observed that EPH alleviated lung oedema and increased the survival rate of ALI mice induced by LPS. Furthermore, EPH administration resulted in a reduction of immune cells and inflammatory cytokines in the lungs of ALI mice. These findings suggest that EPH exerts its protective effect by modulating the immune response and reducing the pulmonary inflammation burden. In our later experiments, we discovered that EPH treatment led to downregulation of the deubiquitinase OTUB1 and increased K48 ubiquitination‐mediated degradation of HIF1α in lung tissues of ALI mice. This finding implies that these proteins may be involved in the molecular pathway underlying the protective effect of EPH on ALI.

ALI can be classified into several types in clinical practice. From the perspective of infection caused by bacteria, viruses, fungi, etc., it can be classified as infectious ALI. From the factors of trauma, surgery and injury, it can lead to ALI. In addition, there are diffuse alveolar damage and ALI caused by exposure to toxins and drugs [42]. In order to investigate the underlying mechanisms of ALI, various animal models have been utilised in current scientific research. Among them, intranasal administration of LPS, direct injection of LPS into the lungs, or inoculation of pathogens in mice are used to simulate inflammation and lung tissue damage caused by ALI. Additionally, connecting mice to a ventilator and subjecting them to high‐pressure ventilation or low tidal volume ventilation is used to simulate lung injury caused by mechanical ventilation. These different types of ALI animal models exhibit distinct characteristics in terms of inflammatory response, oxidative stress, cell apoptosis and the repair process [41, 43, 44]. In the pathogenesis of ALI, the inflammatory response plays a crucial role. Inflammatory mediators such as tumour necrosis factor‐alpha (TNF‐α), interleukin‐1 beta (IL‐1β) and interleukin‐6 (IL‐6) promote the activation of vascular endothelial cells and infiltration of inflammatory cells, leading to inflammation‐induced lung tissue damage [45]. Additionally, the generation of reactive oxygen species (ROS) and nitric oxide (NO), among other oxidants, increases and exceeds the capacity of the body's antioxidant defence system, resulting in further oxidative damage and worsening of the inflammatory response [46, 47]. Similar to these studies, in our research, the presence of LPS caused lung oedema, abnormal alveolar spaces, increased immune cells such as pulmonary macrophages and neutrophils, and elevated levels of inflammatory cytokines IL‐1β, TNF‐α, and IL‐6. Consequently, this led to a decrease in the survival rate of mice.

Current research on OTUB1 mainly focuses on the field of cancer. Studies have shown that OTUB1 can deubiquitinate the MYC protein, making it a potential target for breast cancer treatment [48]. Additionally, OTUB1 can regulate the stability of PD‐L1, thereby influencing cancer immune evasion [49]. In gastric cancer, both CST1 and OTUB1 are involved in the development and progression of the disease [50]. The key point highlighted in these studies is the function of OTUB1 as a deubiquitinase. It can regulate the degradation of various proteins, including YAP, c‐Maf, mTORC1 and HIF1α, thereby mediating cancer development and hypoxic damage in the body or cells [40, 51, 52, 53]. The stability and transcriptional activity of HIF1α are regulated by oxygen levels [54]. Under normal oxygen supply, HIF1α is ubiquitinated and degraded by the von Hippel–Lindau tumour suppressor (VHL) protein complex. In a low oxygen environment, ubiquitination of HIF1α is inhibited, leading to its stabilisation and translocation into the nucleus. In the nucleus, HIF1α forms a complex with HIF1β, binds to specific DNA sequences and activates a series of target genes involved in angiogenesis, metabolic regulation and inflammatory response [55, 56]. Recent studies have shown that HIF1α plays a multifaceted role in ALI. It participates in the regulation of inflammatory cytokine production and activation of inflammatory cells, maintains lung tissue integrity by modulating the function of alveolar epithelial cells and lung interstitial cells, and promotes lung vascular regeneration and repair. OTUB1 can stabilise the HIF1α protein [57]. However, there is limited research on the promoting or inhibitory effects of OTUB1 and HIF1α on the progression of ALI. In our results, as a deubiquitinase, OTUB1 naturally participates in the regulation of this process. In an LPS‐induced ALI animal model, the expression level of OTUB1 significantly increased. Additionally, the expression of OTUB1 was positively correlated with HIF1α, suggesting that OTUB1 may be involved in the regulation of HIF1α in ALI. Furthermore, our subsequent experimental studies revealed that OTUB1 can directly interact with HIF1α and remove ubiquitination modifications on HIF1α. Overexpression of OTUB1 increased the stability of HIF1α. This indicates that OTUB1 affects the stability of HIF1α in ALI by regulating its ubiquitination modifications.

In traditional Chinese medicine, EPH is recognised as one of the main active components of Chinese Ephedra Herb. It has the functions of dispelling wind‐cold, eliminating dampness and resolving phlegm. In Western medicine, this herb is also used for the treatment of respiratory system diseases [14]. In scientific research, some studies have indicated that EPH can be used as an adjunctive therapy for bronchial asthma. It acts on β2‐adrenergic receptors, dilates the bronchi and alleviates symptoms and bronchospasms in asthma patients [58]. Furthermore, EPH promotes the secretion of IL‐10 and inhibits pro‐inflammatory cytokines after LPS stimulation, protecting mice from LPS‐induced injury [19]. In a study on pulmonary fibrosis (EMT), EPH was found to inhibit the EMT process and alleviate bleomycin‐induced lung fibrosis by blocking the NF‐κB signalling pathway and activating the Nrf‐2 signalling pathway [13]. Additionally, EPH treatment can alleviate apoptosis, inflammation and oxidative stress by blocking endoplasmic reticulum stress, thereby relieving chronic obstructive pulmonary disease in vitro and in vivo [21]. While these studies focus on the therapeutic effects and mechanisms of EPH in respiratory system diseases, there is limited knowledge regarding its potential molecular mechanisms in ALI, especially its involvement in regulating hypoxia signalling.

**FIGURE 6:**
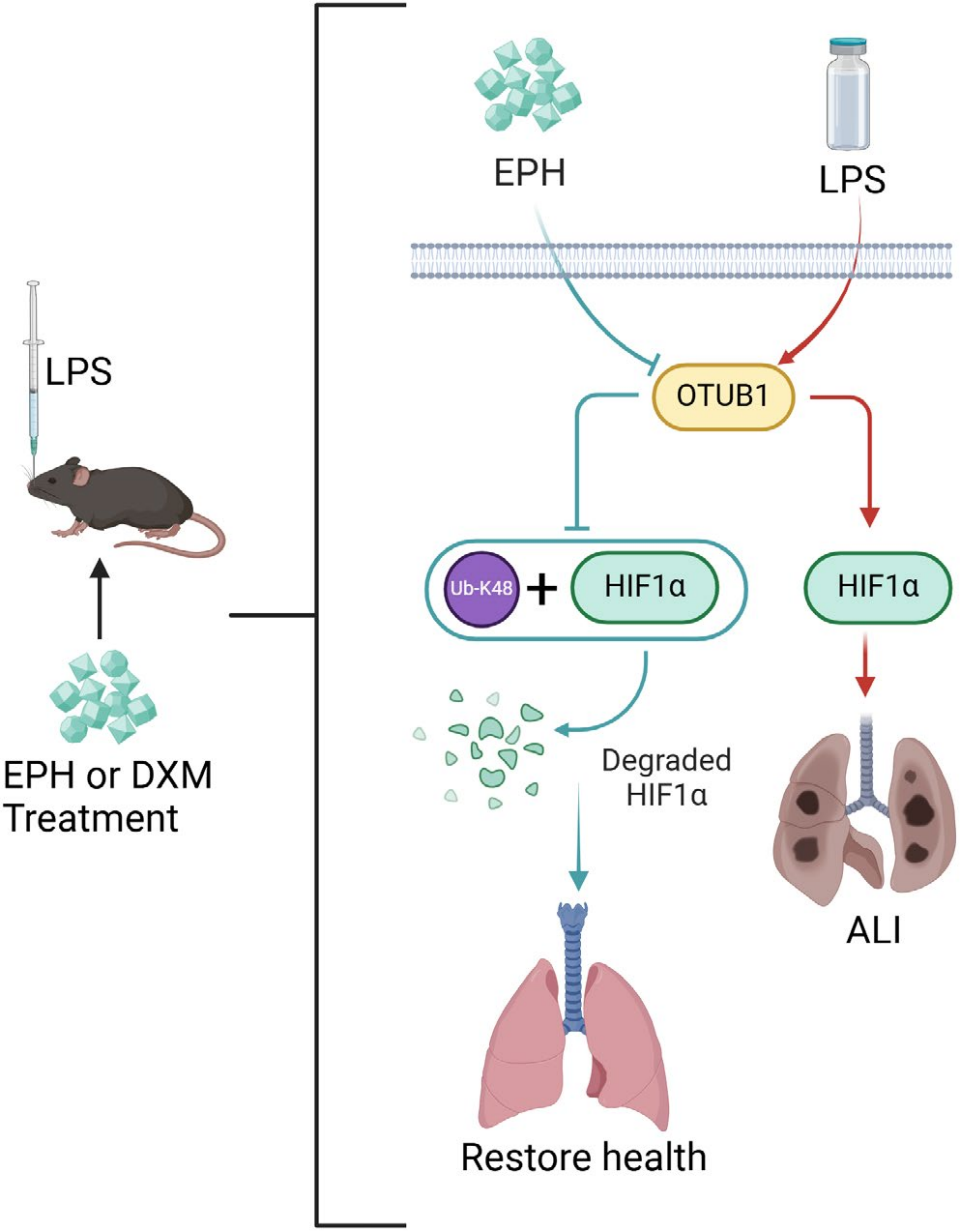
Ephedrine attenuates LPS‐induced acute lung injury in mice by Inhibiting OTUB1 and promoting K48 ubiquitination of HIF1α.

This study provides the first evidence that EPH alleviates ALI symptoms by inhibiting the expression of OTUB1 and promoting the K48 ubiquitination of HIF1α (Figure 6). These findings offer a theoretical foundation and practical experience for understanding the potential signalling pathways involved in EPH treatment for ALI.

## Author Contributions

**Bo Zhou:** conceptualization (lead), data curation (lead), methodology (lead), project administration (lead). **Keke Zhao:** data curation (supporting), formal analysis (lead), software (supporting), writing – original draft (lead), writing – review and editing (lead). **Jiahui Xue:** conceptualization (supporting), data curation (supporting), formal analysis (supporting). **Fangling Zhou:** conceptualization (supporting), software (supporting). **Jin‐ao Duan:** conceptualization (supporting), formal analysis (lead), methodology (lead). **Yang Niu:** funding acquisition (lead), resources (lead). **Hanqing Wang:** funding acquisition (lead), investigation (lead), resources (lead).

## Ethics Statement

All study protocols were conducted in accordance with ethical guidelines and authorised by Ningxia Medical University Medical Ethical Committee (No. 2020‐523).

## Conflicts of Interest

The authors declare no conflicts of interest.

## Data Availability

All data generated from this study, including raw data, is available from the corresponding author on reasonable request.
